# A naturally hypersensitive glucocorticoid receptor elicits a compensatory reduction of hypothalamus–pituitary–adrenal axis activity early in ontogeny

**DOI:** 10.1098/rsob.150193

**Published:** 2016-07-20

**Authors:** Eduard Muráni, Siriluck Ponsuksili, Alexandra Jaeger, Andreas Görres, Armin Tuchscherer, Klaus Wimmers

**Affiliations:** 1 Genome Biology, Leibniz Institute for Farm Animal Biology (FBN), Wilhelm-Stahl-Allee 2, 18196 Dummerstorf, Germany; 2 Genetics and Biometry, Leibniz Institute for Farm Animal Biology (FBN), Wilhelm-Stahl-Allee 2, 18196 Dummerstorf, Germany

**Keywords:** glucocorticoid receptor, gain-of-function mutation, hypothalamus–pituitary–adrenal axis, glucocorticoids, corticotropin-releasing hormone, corticosteroid-binding globulin

## Abstract

We comprehensively characterized the effects of a unique natural gain-of-function mutation in the glucocorticoid receptor (GR), GR_Ala610Val_, in domestic pigs to expand current knowledge of the phenotypic consequences of GR hypersensitivity. Cortisol levels were consistently reduced in one-week-old piglets, at weaning and in peripubertal age, probably due to a reduced adrenal capacity to produce glucocorticoids (GC), which was indicated by an adrenocortical thinning in GR_Ala610Val_ carriers. Adrenocorticotrophic hormone (ACTH) levels were significantly reduced in one-week-old piglets only. Expression analyses in peripubertal age revealed significant downregulation of hypothalamic expression of *CRH* and *AVP*, the latter only in females, and upregulation of hepatic expression of *SERPINA6*, by GR_Ala610Val_. Transcriptional repression of proinflammatory genes in peripheral blood mononuclear cells (PBMCs) from GR_Ala610Val_ carriers was more sensitive to dexamethasone treatment *ex vivo*. However, no significant effects on growth, body composition, blood chemistry or cell counts were observed under baseline conditions. These results suggest that GR_Ala610Val_-induced GR hypersensitivity elicits a compensatory reduction in endogenous, bioactive glucocorticoid levels via readjustment of the hypothalamus–pituitary–adrenal (HPA) axis early in ontogeny to maintain an adequate response, but carriers are more sensitive to exogenous GC. Therefore, GR_Ala610Val_ pigs represent a valuable animal model to explore GR-mediated mechanisms of HPA axis regulation and responses to glucocorticoid-based drugs.

## 1. Introduction

The glucocorticoid receptor (GR) is a member of the nuclear receptor superfamily of ligand-activated transcription factors. GR is ubiquitously expressed, and it is the primary mediator of the effects of glucocorticoids (GC) on a variety of physiological functions, including energy metabolism and immune responses [1]. Activated GRs repress the expression of a large set of proinflammatory genes [2,3]. Therefore, GR ligands, such as dexamethasone (DEX), have broad application in the treatment of inflammatory diseases and autoimmune disorders, and the ligands are among the most widely prescribed drugs in human and veterinary medicine [1,4]. However, the therapeutic use of GC-based drugs is limited by possible adverse side effects (e.g. osteoporosis [5]) and highly variable individual responses [6]. GRs are key players in the feedback regulation of the hypothalamus–pituitary–adrenal (HPA) axis and the orchestration of the stress response [7]. Impaired GR signalling may lead to maladaptive stress responses, which is implicated in the pathogenesis of stress-related psychiatric disorders in humans, such as major depression [8]. Diverse factors may influence GR signalling, including genetic and epigenetic alterations [9], and knowledge of these effects promises to improve GC therapy and prevent stress-related disorders.

Targeted mutagenesis has provided important insights into the physiological functions and molecular actions of GR, and this technique is an indispensable tool to study the consequences of GR dysregulation. Most functionally and/or phenotypically characterized artificial and natural mutations cause GR deficiency [10–12]. Notably, GR loss-of-function mutations are associated with different phenotypic consequences, depending on which tissue or GR domain, i.e. signalling pathway, is affected [10,13].

However, little research has been performed on GR hypersensitivity, and the molecular causes and phenotypic consequences of this condition are not well understood [14,15]. Gain-of-function mutations of GRs, particularly in the ligand binding domain (LBD), are very rare [11,15]. The only phenotypically characterized model of a gain-of-function GR mutation is a knock-in of the GR_M610 L_ substitution in mice [16].

We recently discovered a natural alanine to valine substitution in the LBD of the porcine GR (GR_Ala610Val_ induced by SNP c.1829C>T in the porcine *NR3C1* [17]), which causes large inter-individual variations in adrenal weight and stress-induced plasma cortisol levels in domestic pigs [17]. We used an *in vitro* transactivation assay and demonstrated that the GR_Ala610Val_ substitution increased GC responsiveness of the porcine GR. The more GC-sensitive Val variant was associated with markedly lower adrenal weight and cortisol levels in slaughter blood. The GR_Ala610Val_ substitution, which occurred at a position directly adjacent to the site mutated by Zhang *et al*. [16], is the only known natural gain-of-function mutation of the GR-LBD. In this study, we exploited this unique animal model to comprehensively characterize the phenotypic consequences of GR hypersensitivity in a natural setting. We investigated the effect of the GR_Ala610Val_ substitution on different aspects of HPA axis function during ontogeny, including basal and stress-induced secretion of cortisol and adrenocorticotrophic hormone (ACTH), and HPA axis regulation at the hypothalamic level. We also analysed the effect of the GR_Ala610Val_ substitution on different GC-modulated traits related to growth, body composition and immune responses. We examined the expression of GR target genes and GR signalling pathways *in vivo* in relevant tissues and *ex vivo* in DEX-treated peripheral blood mononuclear cells to gain insights into the molecular and physiological mechanisms of GR action.

## 2. Results

### 2.1. The GR_Ala610Val_ substitution does not negatively impact carrier viability

Two German Landrace sires heterozygous for the GR_Ala610Val_ substitution were each mated to five different heterozygous German Landrace dams, which gave birth to a total of 10 litters with 104 offspring (43 female and 61 male piglets in total). Genotype distribution in the offspring (AlaAla : AlaVal : ValVal = 22 : 51 : 27 per cent) was not significantly different from the expected 25 : 50 : 25 proportion (*p* = 0.480). Birth weight was not significantly influenced by the GR_Ala610Val_ substitution or by GR_Ala610Val_ × sex (G × S) interaction (electronic supplementary material, table S1). These findings suggest that the GR_Ala610Val_ substitution did not negatively affect carrier viability.

### 2.2. Effect of the GR_Ala610Val_ substitution on basal and stress-induced cortisol and ACTH levels

**Figure 1.**
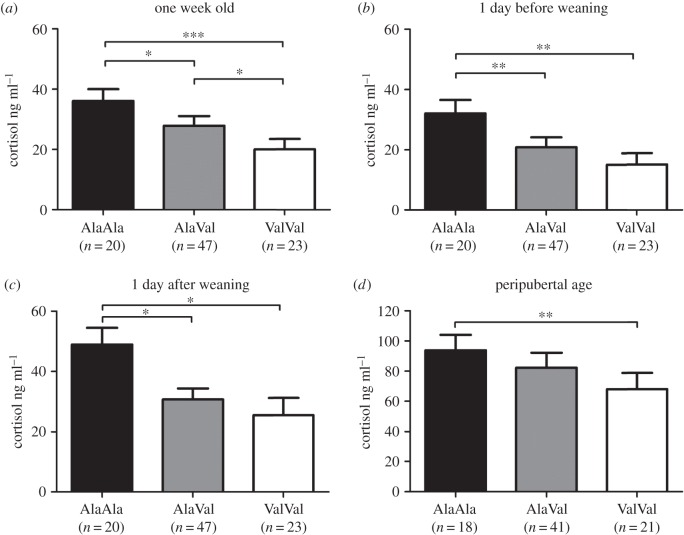
Effect of the GR_Ala610Val_ substitution on plasma cortisol levels. (*a*) Basal levels in one-week-old piglets; (*b*) basal levels one day before weaning; (*c*) stress-induced levels one day post-weaning; (*d*) stress-induced levels in peripubertal pigs. Data are presented as least-squares means ± s.e. *p < 0.05, **p < 0.01, ***p < 0.001.

**Figure 2.**
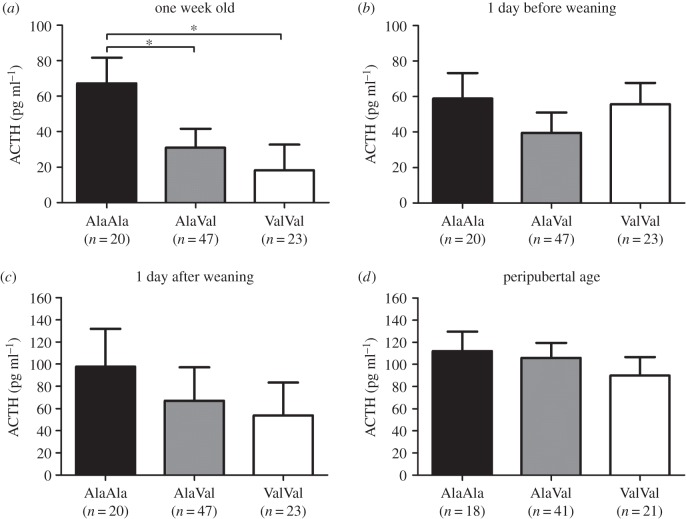
Effect of the GR_Ala610Val_ substitution on plasma ACTH levels. (*a*) Basal levels in 1-week-old piglets; (*b*) basal levels 1 day before weaning; (*c*) stress-induced levels 1 day post-weaning; (*d*) stress-induced levels in peripubertal pigs. Data are presented as least-squares means ± s.e. *p < 0.05.

We analysed basal plasma cortisol and ACTH levels one week *post natum* (pn) and 1 day before weaning (bw) and stress-induced levels 1 day after weaning (aw) and at a peripubertal age in slaughter blood to examine whether the effect of the GR_Ala610Val_ substitution on HPA axis activity depended on age and/or animal state. All blood samplings were performed in the morning at the peak of HPA axis activity. The GR_Ala610Val_ genotype produced a significant effect on both parameters (*p* < 0.001 for cortisol and *p* = 0.015 for ACTH) in one-week-old piglets. Piglets carrying the Val variant exhibited significantly lower basal plasma cortisol and ACTH levels compared with homozygous wild-type AlaAla piglets (figures 1*a* and 2*a*; electronic supplementary material, table S1). Basal cortisol (*p* = 0.001), but not ACTH (*p* = 0.077), levels were significantly reduced by the GR_Ala610Val_ substitution one day before weaning (four weeks pn) (figures 1*b* and 2*b*; electronic supplementary material, table S1). Weaning pigs from the sow is one of the most stressful events in a pig's life [18]. Accordingly, mean plasma cortisol and ACTH levels across all animals were significantly elevated 1 day after weaning (mean cortisol level increased from 18.7 ng ml^−1^ bw to 34.0 ng ml^−1^ aw, *p* < 0.001; mean ACTH level increased from 43.4 pg ml^−1^ bw to 75.5 pg ml^−1^ aw, *p* < 0.001). Although the relative change in cortisol levels between the two time points was similar in all three genotype groups, piglets carrying the Val variant exhibited significantly lower cortisol levels 1 day post-weaning compared with wild-type piglets (genotype *p* = 0.008; figure 1*c*; electronic supplementary material, table S1). Carriers of the Val variant also displayed lower ACTH levels compared with wild-type animals 1 day after weaning, but this difference did not reach statistical significance (genotype *p* = 0.111; figure 2*c*; electronic supplementary material, table S1). The Val variant was associated with significantly lower cortisol levels in slaughter blood (genotype *p* = 0.008), which is consistent with our previous study [17], but ACTH levels in carriers of the Val variant were non-significantly reduced compared with plasma ACTH levels of wild-type individuals (genotype *p* = 0.543; figures 1*d* and 2*d*; electronic supplementary material, table S1). Repeated measures analysis across all four sampling time points confirmed an overall trend of the GR_Ala610Val_ substitution to reduce plasma cortisol (genotype *p* < 0.001; electronic supplementary material, table S1) and ACTH levels (genotype *p* = 0.001; electronic supplementary material, table S1), which were approximately 40–50% lower in homozygous carriers of the Val variant compared with wild-type littermates.

### 2.3. The GR_Ala610Val_ substitution influences adrenal cortex, but not adrenal medulla, size

**Figure 3.**
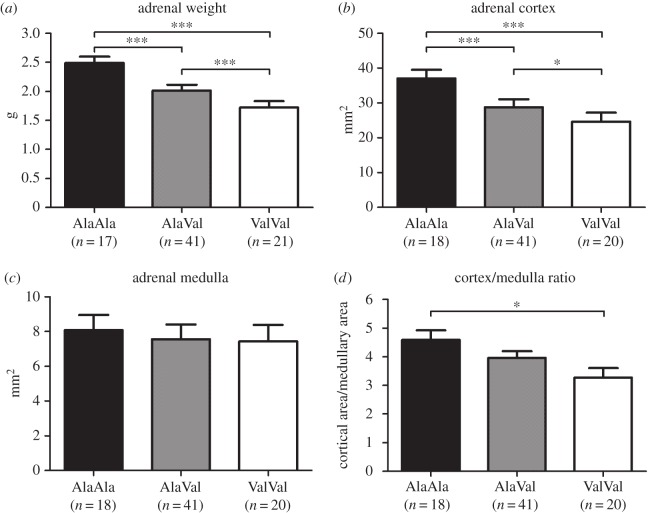
Effect of the GR_Ala610Val_ substitution on weight and structure of the adrenal gland (left). (*a*) Adrenal weight; (*b*) cross-sectional area of the adrenal cortex; (*c*) cross-sectional area of the adrenal medulla; (*d*) cortex/medulla ratio calculated using their cross-sectional areas. Data are presented as least-squares means ± s.e. *p < 0.05, **p < 0.01, ***p < 0.001.

We found a markedly reduced weight of the adrenal glands in carriers of the Val variant (genotype *p* < 0.001; figure 3*a*; electronic supplementary material, table S1), which is consistent with the observed persistent reduction of cortisol production caused by the GR_Ala610Val_ substitution and our previous findings [17]. We performed histological analyses of both adrenal glands (only results for the left adrenal are presented here) to investigate how the reduction in weight was reflected in adrenal structure. The results revealed a similar marked, highly significant reduction in size of the adrenal cortex associated with the Val variant (genotype *p* < 0.001; figure 3*b*; electronic supplementary material, table S1), but only a slight and insignificant reduction in the size of the adrenal medulla (genotype *p* = 0.639; figure 3*c*; electronic supplementary material, table S1). These changes resulted in a significantly reduced cortical/medullary ratio in carriers of the Val variant (genotype *p* = 0.022; figure 3*d*; electronic supplementary material, table S1). The results were similar for both adrenal glands (electronic supplementary material, table S1). We wondered if the production of other corticosteroids was affected by the pronounced adrenocortical thinning in carriers of the GR_Ala610Val_ substitution. Therefore, we analysed plasma aldosterone levels in slaughter blood. We found no significant effect of the GR_Ala610Val_ substitution (genotype *p* = 0.410; electronic supplementary material, table S1), which indicates that the effect of the substitution was limited to glucocorticoid production.

Analysis of pituitary weight revealed only a slight, insignificant, reduction in homozygous ValVal animals (genotype *p* = 0.144; electronic supplementary material, table S1).

### 2.4. Hypothalamic expression of corticotropin-releasing hormone and vasopressin is downregulated by the GR_Ala610Val_ substitution

**Figure 4.**
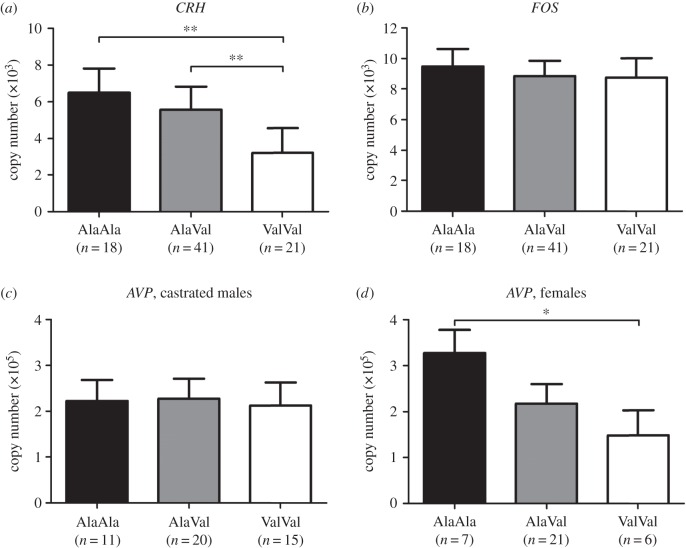
Effect of the GR_Ala610Val_ substitution on gene expression in hypothalamus of peripubertal pigs. (*a*) *CRH*; (*b*) *FOS*; (*c*) *AVP* in castrated males; (*d*) *AVP* in intact females. Gene expression was measured using quantitative real time PCR. Data are presented as least-squares means ± s.e. *p < 0.05, **p < 0.01.

We measured the mRNA expression of the two main ACTH secretagogues, corticotropin-releasing hormone (*CRH*) and vasopressin (*AVP*), in samples collected from peripubertal pigs using quantitative real time PCR (qPCR) to examine the impact of the GR_Ala610Val_ substitution on HPA axis regulation in the hypothalamus. The results demonstrated that *CRH* expression was significantly downregulated by the GR_Ala610Val_ substitution (genotype *p* = 0.002; figure 4*a*; electronic supplementary material, table S1). Notably, the effect of the GR_Ala610Val_ substitution on *AVP* expression depended on carrier gender (G × S interaction, *p* = 0.045). Hypothalamic *AVP* expression was significantly downregulated by the GR_Ala610Val_ substitution in females (figure 4*d*), whereas castrated males (all male piglets were castrated 4 days pn) exhibited no differences in *AVP* expression between the different genotype groups (figure 4*c*; electronic supplementary material, table S1). We measured the mRNA expression of c-fos (*FOS*), which is a marker of neuronal activation following stress [19]. We found no significant effect of the GR_Ala610Val_ substitution (genotype *p* = 0.823) on *FOS* expression (figure 4*b*). This finding suggests that the observed differences in *CRH* and *AVP* expression between the different genotype groups of GR_Ala610Val_ were not caused by their differential reactivity to slaughter-associated stress.

### 2.5. The GR_Ala610Val_ substitution does not significantly affect growth, body composition, blood chemistry or cell counts under baseline conditions

The GR is involved in the regulation of energy homeostasis and immune responses [1]. Therefore, we analysed effect of the GR_Ala610Val_ substitution on different related trait complexes (electronic supplementary material, table S1), including growth during the fattening period (average daily gain, average daily feed intake), body composition in peripubertal age (average backfat thickness, area of *M. longissimus dorsi*, estimated lean content, carcass length), blood chemistry (glucose, total cholesterol, triglyceride and blood urea nitrogen levels before weaning and in peripubertal age), and blood cell counts (leucocyte number, lymphocyte and neutrophil percentage and their ratio at all four blood sampling points). We found no significant effects of the GR_Ala610Val_ substitution on any of the analysed traits, except leucocyte number after weaning (genotype *p* = 0.002; electronic supplementary material, table S1).

### 2.6. Expression of *SERPINA6*, which encodes corticosteroid-binding globulin, is upregulated by the GR_Ala610Val_ substitution in liver

The apparent absence of detectable phenotypic consequences of the GR_Ala610Val_ substitution, except on HPA axis function, suggested that potential compensatory mechanisms balanced cortisol production and tissue responses to GC. Therefore, we analysed the expression of selected genes in hypothalamus (representing HPA axis tissues) and liver (representing peripheral tissues) sampled from peripubertal pigs using qPCR. These genes included GR targets (*GILZ*; *BDNF* and *PCK1* in hypothalamus and liver, respectively), genes from the GC signalling pathway (*NR3C1*, *NR3C2*, *FKBP4* and *FKBP5*), and genes regulating the bioavailability of GC at the tissue level (*HSD11B1* and *HSD11B2*) and systemic level (*SERPINA6*, *SRD5A1* and *AKR1D1* in liver only).

We found no significant effects of the GR_Ala610Val_ substitution or the G × S interaction on the expression of selected genes in hypothalamus, including *NR3C1*, which encodes GR, and *NR3C2*, which encodes the mineralocorticoid receptor (electronic supplementary material, table S1).

**Figure 5.**
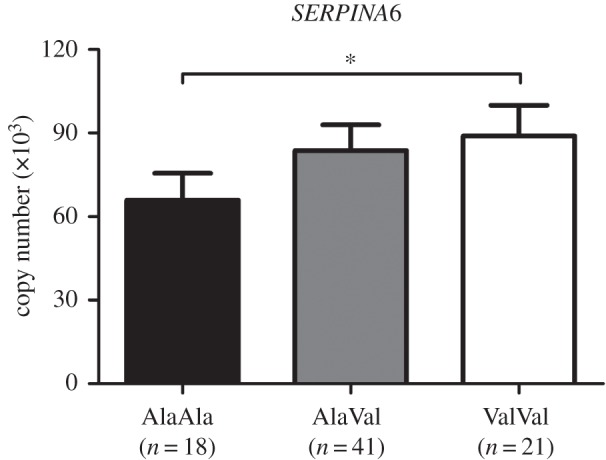
Effect of the GR_Ala610Val_ substitution on expression of *SERPINA6*, which encodes corticosteroid-binding globulin, in liver of peripubertal pigs. *SERPINA6* expression was measured using quantitative real time PCR. Data are presented as least-squares means ± s.e. *p < 0.05.

We found significant effects of the GR_Ala610Val_ substitution on the hepatic expression of *FKBP4* (genotype *p* = 0.018) and *SERPINA6* (genotype *p* = 0.034; figure 5), which encodes the corticosteroid-binding globulin (CBG), which is a major regulator of the systemic bioavailability and clearance of GC [20]. Both genes were upregulated in carriers of the Val variant (electronic supplementary material, table S1). We also found a significant effect of a G × S interaction on the expression of *HSD11B1* in liver (*p* = 0.020), but there was no clear direction of the effect (electronic supplementary material, table S1).

We further analysed the expression of genes from the CRH signalling pathway (*CRHR1*, *CRHR2* and *CRHBP*) in hypothalamus, but we found no significant effect of the GR_Ala610Val_ substitution (electronic supplementary material, table S1).

### 2.7. Glucocorticoid sensitivity of peripheral blood mononuclear cells is enhanced by the GR_Ala610Val_ substitution *ex vivo*

**Figure 6.**
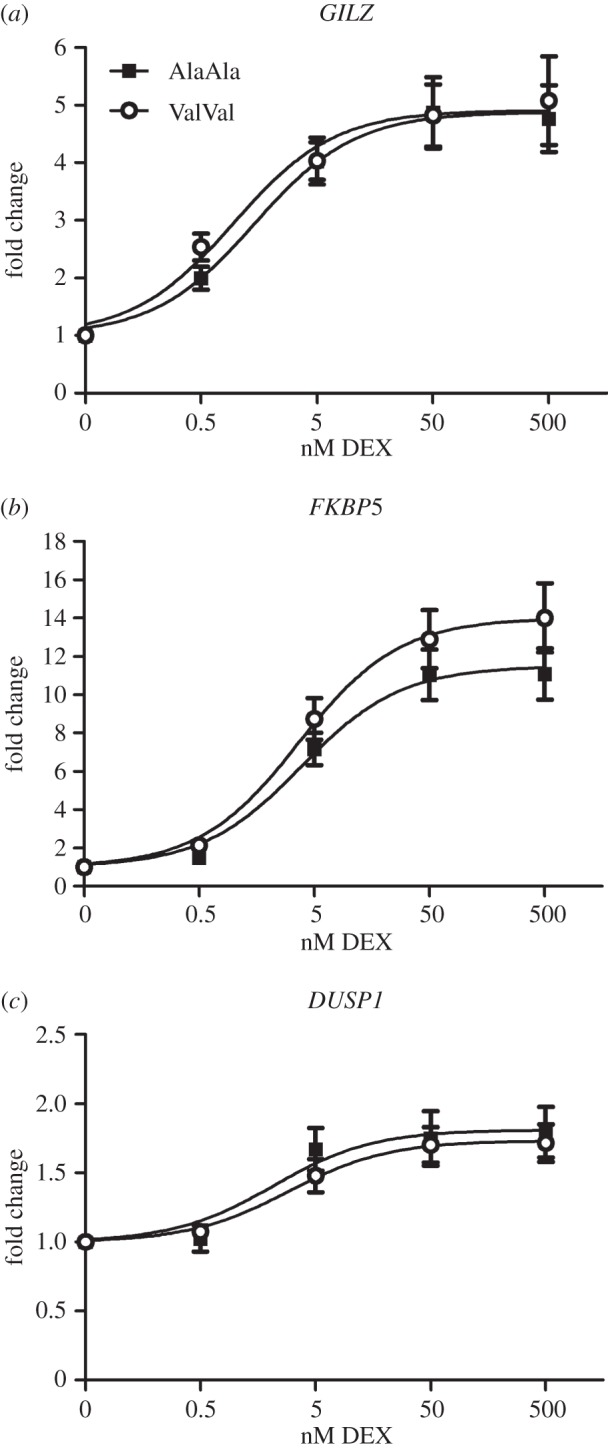
Transcriptional activation in ConA-stimulated PBMCs in response to DEX treatment *ex vivo*: (*a*) *GILZ*; (*b*) *FKBP5*; (*c*) *DUSP1*. Gene expression was measured using quantitative real time PCR. Data are presented as dose–response curves estimated based on fold-changes calculated within each individual relative to control treatment with ConA but without DEX. Individual points show means ± s.e. Estimated half-maximal effective concentrations (EC50) for AlaAla (*n* = 9) compared to ValVal (*n* = 10) carriers were: (*a*) 1.445 versus 0.957 nM (*p* = 0.561) for *GILZ*; (*b*) 3.857 versus 3.686 nM (*p* = 0.932) for *FKBP5*; (*c*) 2.156 versus 2.948 nM (*p* = 0.932) for *DUSP1*.

**Figure 7.**
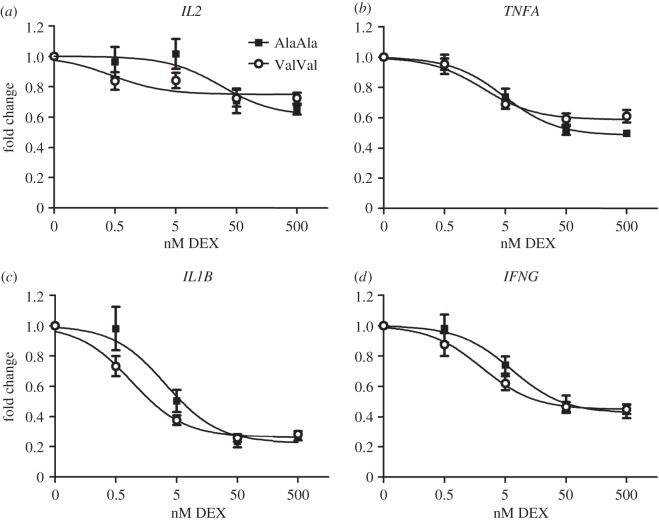
Transcriptional repression in ConA-stimulated PBMCs in response to DEX treatment *ex vivo*: (*a*) *IL2*; (*b*) *TNFA*; (*c*) *IL1B*; (*d*) *IFNG*. Gene expression was measured using quantitative real time PCR. Data are presented as dose–response curves estimated based on fold-changes calculated within each individual relative to control treatment with ConA but without DEX. Individual points show means ± s.e. Estimated half-maximal inhibitory concentrations (IC50) for AlaAla (*n* = 9) compared to ValVal (*n* = 10) carriers were: (*a*) 28.35 versus 0.442 nM (*p* = 0.006) for *IL2*; (*b*) 4.791 versus 2.151 nM (*p* = 0.076) for *TNFA*; (*c*) 6.331 versus 2.063 nM (*p* = 0.042) for *IL1B;* (*d*) 3.330 versus 0.911 nM (*p* = 0.004) for *IFNG*.

Circulating cortisol levels are reduced by the GR_Ala610Val_ substitution *in vivo*. Therefore, we examined the effect of the GR_Ala610Val_ substitution on tissue responses to equally strong GC stimuli. We analysed the transcriptional response of glucocorticoid-induced (*GILZ*, *FKBP5*, *DUSP1*) and -repressed (*IL2*, *TNFA*, *IFNG*, *IL1B*) genes [2,3] to different DEX concentrations (0, 0.5, 5, 50 and 500 nM) in mitogen-stimulated (concanavalin A, ConA) peripheral blood mononuclear cells (PBMCs) *ex vivo* in cell culture. None of the genes exhibited significant differential expression associated with the GR_Ala610Val_ substitution in the absence of DEX (data not shown). Dose–response curves of the transcriptional response of *GILZ* and *FKBP5* to DEX exhibited a notable leftward shift in homozygous ValVal individuals compared with PBMCs of wild-type AlaAla animals (figure 6*a,b*). However, the difference in estimated half-maximal effective concentrations (EC50) was not significant for any of the activated genes (electronic supplementary material, table S2). The estimated half-maximal inhibitory concentration (IC50) for repressed genes was significantly lower in PBMCs of homozygous ValVal animals compared with PBMCs of wild-type AlaAla animals for *IL2* (*p* = 0.006), *IFNG* (*p* = 0.042) and *IL1B* (*p* = 0.004) (figures 7*a,c,d*; electronic supplementary material, table S2). The reduction in the IC50 of *TNFA* associated with GR_Ala610Val_ approached statistical significance (*p* = 0.076, figure 7*b*; electronic supplementary material, S2). These results demonstrated that proinflammatory response of PBMCs of carriers of the GR_Ala610Val_ substitution was more potently repressed by exogenous GC and confirmed the gain-of-function character of this mutation.

## 3. Discussion

Given the widespread expression of GR and its involvement in diverse biological processes, GR_Ala610Val_ could potentially influence a broad spectrum of physiological functions. However, we found essentially no phenotypic manifestations of the substitution on growth, body composition, blood chemistries, including glucose, cholesterol and triglyceride levels, or blood cell counts, except for the approximately 40–50% reduction in HPA axis activity. Nevertheless, we found significantly lower leucocyte numbers 1 day after weaning in carriers of the substitution, which suggests that the effect of GR_Ala610Val_ is revealed by exposure of the animals to certain stressors.

The apparent absence of phenotypic consequences of the GR_Ala610Val_ substitution under baseline conditions is supported by findings in the mouse GR hypersensitivity model generated using a knock-in of the GR_M610 L_ substitution [16] (corresponding mutated Met residue is at position 609 in porcine GR). Mice carrying the gain-of-function allele displayed no obvious phenotypic abnormalities in serum electrolyte, glucose or lipid levels, bone mineral density, serum testosterone levels, or percentage body fat compared with wild-type littermates, but markedly reduced HPA axis activity was observed, which is similar to the natural pig model [16]. Specifically, adult mice expressing the gain-of-function mutation exhibited reduced basal and stress-induced corticosterone and ACTH levels and adrenocortical size [16]. These results suggest that enhanced GR sensitivity due to structural mutations in the LBD induces a compensatory decrease in GC levels via an enhanced negative feedback regulation of the HPA axis to maintain an adequate GC response. This study found a significant reduction in basal cortisol and ACTH levels in carriers of the GR_Ala610Val_ substitution as early as one week *post natum*. This finding indicates that readjustment of the HPA axis activity due to GR hypersensitivity starts early in ontogeny. Examination of HPA axis activity during prenatal development in GR-deficient mice revealed that the feedback regulation of GC production is established during the fetal stage at approximately day E16.5 [21]. We also found significantly lower basal and post-stress cortisol levels in carriers of the GR_Ala610Val_ substitution later in ontogeny. However, the effect of the GR_Ala610Val_ substitution on basal and post-stress ACTH levels became subtle at weaning and puberty. Targeted disruption of GR in the pituitary (GR^POMCCre^) [22] and the paraventricular nucleus (PVN) of the hypothalamus (Sim1Cre-GRe3delta) [23] in mice revealed a key role of pituitary GR in the regulation of the HPA axis in early postnatal development, with PVN GR picking up in adolescence through adulthood [23]. Therefore, the effect of the GR_Ala610Val_ substitution on ACTH level may be age-dependent. Notably, diverse models of GR deficiency (GR^NesCre^; Sim1Cre-GRe3delta) [24,25] and a model of GR excess induced by GR overexpression (YGR) [26] exhibited a dissociation of GC and ACTH levels. A persistent reduction in cortisol production is likely in carriers of the GR_Ala610Val_ substitution, which may be at least partially explained by the reduced capacity of the adrenal cortex to produce GC, which was indicated by its markedly reduced size. GR_M610 L_ mice exhibited significantly lower corticosterone secretion even in response to ACTH stimulation [16], which provides supporting evidence for this conclusion. By contrast, the production of other corticosteroids was not affected, as evidenced by the lack of effect of the GR_Ala610Val_ substitution on aldosterone levels, which implies a specific influence of the GR_Ala610Val_ substitution on *zona fasciculata* cells. It remains to be elucidated whether the reduced size of the adrenal cortex was due to reduced ACTH levels earlier in ontogeny or whether it involves local GR effects in the adrenal gland [27] or its pre/perinatal development.

We found a significantly reduced expression of *CRH* and *AVP* in the hypothalamus of carriers of the gain-of-function variant at a peripubertal age, which is consistent with the hypothesis that PVN GR plays an important role in HPA axis regulation from early adolescence onward. Notably, the effect of the GR_Ala610Val_ substitution on *AVP* expression only occurred in females. Sexual dimorphism in the regulation of *AVP* expression in rat PVN by GC was demonstrated, with a more pronounced downregulation of *AVP* in females [28]. However, we found no significant effect of the GR_Ala610Val_ × sex interaction on ACTH or cortisol levels. Vasopressin alone is a relatively weak stimulus for ACTH secretion [29]; therefore, the downregulation of *CRH* expression may be sufficient to reduce ACTH and cortisol levels in castrated males carrying the gain-of-function variant. CRH and AVP are not only central drivers of the HPA axis, but they also act as neuromodulators in the brain and affect among others higher mental functions, including emotion, cognition and behaviour [8]. We examined the expression of several genes from the CRH signalling pathway (*CRHR1*, *CRHR2*, *CRHBP*) in hypothalamus and found no compensatory changes associated with GR_Ala610Val_. Consequently, although the compensatory downregulation of GC levels probably alleviates some effects of GR hypersensitivity due to the GR_Ala610Val_ substitution, this may still influence brain functions that are regulated by CRH and AVP independently of the HPA axis, for example behavioural responses to stress [30]. Notably, atypical depression shows similar features to HPA axis function in GR_Ala610Val_ pigs. Atypical depression is characterized by centrally mediated hypoactivity with low CRH levels, hypocorticolism and increased GC sensitivity, and it exhibits a markedly higher prevalence in women than men [31,32].

We also found no significant compensatory changes in the expression of several genes regulating GC action, including the GR gene *NR3C1* itself, in the hypothalamus. By contrast, we found a significant upregulation of *SERPINA6* in the liver of carriers of the gain-of-function allele. CBG, encoded by *SERPINA6*, is a major regulator of the systemic bioavailability of GC, and it sequesters approximately 80% of circulating GC in an inactive complex [20]. Therefore, upregulation of *SERPINA6* should reduce free, biologically active GC and consequently dampen tissue GC responses, which may be an additional compensation mechanism for GR hypersensitivity caused by the GR_Ala610Val_ substitution. It is likely that hepatic expression of *SERPINA6* was readjusted earlier in ontogeny, similar to HPA axis activity. Increased GR activation in early ontogeny is able to induce concomitant readjustments of HPA axis activity and CBG secretion, which was evidenced by lower basal plasma cortisol and increased CBG concentrations in the offspring of sows stressed during gestation [33] or in neonatal pigs subjected to stressful handling [34]. Further research is needed to explain the molecular mechanisms and functional significance of *SERPINA6* upregulation by the GR_Ala610Val_ substitution.

To examine the impact of GR_Ala610Val_ on gene expression in the absence of the systemic feedback regulation of GC levels, we analysed transcriptional response of several GR target genes in mitogen-stimulated PBMCs to DEX *ex vivo.* In this context, the effect of the substitution seems to be dependent on the overall responsiveness of the target gene expression to DEX and on the mode of its regulation by GR. GR regulates gene expression essentially either directly by binding to cognate response elements (GRE) in regulatory regions of target genes, or indirectly via interactions with other transcription factors and co-regulators [35,36]. The effect of GR_Ala610Val_ was most pronounced for indirectly repressed genes (e.g. *IL1B*), while genes whose expression is directly activated by GR (e.g. *GILZ*) showed no significant differences. Until recently, it was generally believed that the therapeutic, anti-inflammatory action of GC is based on protein–protein interaction-dependent repression of inflammatory genes by GR monomers whereas undesirable side effects, for instance on metabolism, are based on direct gene activation by GR homodimers [5]. This model of functional separation of therapeutic and adverse effects initiated intensive efforts to develop GR ligands that could dissociate activation and repression by influencing oligomerization state of GR; however, so far these have met with little success [37,38]. There is mounting evidence that the original model of dissociation between activation and repression of gene expression by GR was oversimplified and that gene repression involves multiple mechanisms [37–39]. In view of the complexity of GR function and its intertwined physiological effects, the mechanism which favours effects of GR_Ala610Val_ on repression over activation of gene expression in mitogen-stimulated PBMC and its relevance for phenotypic consequences of GR_Ala610Val_ is not obvious. Different from this study, our previous *in vitro* analyses using a MMTV-driven reporter showed significantly enhanced transcriptional activity of the valine variant [17]. Hence, the effect of GR_Ala610Val_ on transcriptional response appears to be context-dependent. Recent research suggests that contextual ability of GR to elicit specific transcriptional responses is conferred by the interaction between GR, co-regulators such as GRIP1, and the chromatin landscape [40,41]. There is evidence from the structural analysis of the LBD of the human GR that GR_Ala610Val_ probably influences architecture of the GR-LBD and its interaction with ligands [42,43]. Changes in GR-LBD structure might expose different interacting surfaces and result in differential recruitment of co-regulators [36,44]. Thus, to better understand phenotypic consequences of GR_Ala610Val_, future research should focus on its effect on GR regulatory networks and chromatin landscape in different cellular contexts and activation states.

Taken together, our results suggest that GR hypersensitivity induced by the GR_Ala610Val_ substitution produces a compensatory reduction in bioactive GC levels, but it does not lead to notable alterations in GC-regulated processes and phenotypic traits under baseline conditions. We provide evidence for the first time that this compensation occurs early in ontogeny and involves *CRH* and *AVP* expression in hypothalamus and *SERPINA6* expression in liver. Our results suggest that the effect of GR_Ala610Val_ appears under stress exposure, particularly given the downregulation of *CRH* and *AVP* in hypothalamus. Carriers of the GR_Ala610Val_ substitution are probably more sensitive to exogenous GC, as suggested by our analysis of the transcriptional response to GC in PBMCs. These features promote the use of pigs carrying the GR_Ala610Val_ substitution as a valuable animal model to investigate the responses to GC-based drugs and explore mechanisms of HPA axis regulation, which may be relevant in the genetic basis of depression.

## 4. Material and methods

### 4.1. Animals and sample collection

The animals (purebred German Landrace pigs) used in this study were born and reared at the experimental pig farm of the Leibniz Institute for Farm Animal Biology (FBN) in Dummerstorf (Germany). Two sires heterozygous for the GR_Ala610Val_ substitution were each bred using artificial insemination to five different heterozygous dams. All piglets (*n* = 104; 43 female and 61 male) were weighed at birth. Weak piglets weighing less than 800 g were excluded from subsequent experiments. This intervention did not alter genotype proportions (electronic supplementary material, table S1). Male piglets were castrated 4 days pn. Piglets were fed ad libitum with standard diets (Trede & von Pein, Itzehoe, Germany) after weaning at four weeks pn. Pigs (*n* = 75; 32 females and 43 male castrates) were housed in pens equipped with an electronic feed dispenser (Insentec, B.V., Marknesse, The Netherlands) that recorded individual feed intake through ear tag transponders and weighed at 10, 15 and 19 weeks pn to measure growth and feed intake during the fattening period. Pigs were slaughtered in the experimental slaughter facility of the FBN, Dummerstorf after reaching a body weight of approximately 105 kg (approx. 22 weeks pn*;* peripubertal age). Animals were exsanguinated following electronarcosis and dissected (*n* = 80; 34 females and 46 male castrates). Phenotypic records, including body composition traits, were obtained according to German performance test directives.

Blood samples (5 ml) were obtained via rapid (less than or equal to 30 s) *anterior vena cava* puncture at the circadian peak of HPA axis activity (08.00) from one-week-old piglets, and 1 day before and 1 day after weaning (*n* = 90; 38 females and 52 male castrates). Trunk blood (50 ml) was collected during exsanguination at slaughter (09.30). Samples were collected into pre-chilled EDTA-containing tubes and stored on ice for plasma and PBMC preparation.

Tissues samples were quickly dissected after slaughter. Pituitary and both adrenal glands were weighed before further processing. Middle portions of both adrenal glands were excised and frozen in isopentane for histological analyses. A stereotaxic atlas of the pig brain [45] served as a reference for dissection of the hypothalamus. Samples for RNA extraction were frozen in liquid nitrogen and stored at −80°C until analysis. A small piece of liver tissue was collected for genomic DNA isolation.

All tissue and blood samples were collected in random order.

### 4.2. Genotyping

The GR_Ala610Val_ substitution (SNP *NR3C1* c.1829C>T) was genotyped using a pyrosequencing assay as described previously [17].

### 4.3. Measurement of blood cell counts, chemistry, hormone levels and peripheral blood mononuclear cell preparation

An aliquot of the collected whole blood was used for blood cell counts using an ABX Pentra 60 device (HORIBA ABX SAS, Montpellier, France). Plasma was prepared by centrifugation for 20 min at 4°C and 2000*g* and stored at −80°C until use. PBMCs were prepared via centrifugation on a Histopaque density gradient (Sigma-Aldrich, Taufkirchen, Germany). PBMCs isolated from slaughter blood were cryopreserved for subsequent cell culture analysis in a freezing medium consisting of 90% fetal bovine serum (FBS; PAA, Cölbe, Germany) and 10% DMSO.

Plasma ACTH, cortisol and aldosterone levels were measured in duplicate using commercially available enzyme-linked immunosorbent assays according to the manufacturer's recommendations (DRG, Marburg, Germany). The assays for ACTH and cortisol were validated for porcine plasma previously ([46] and [17], respectively). The intra- and inter-assay coefficients of variation of the aldosterone assay were both less than 4%.

Plasma levels of glucose, total cholesterol, triglycerides and blood urea nitrogen were measured using a Fuji DriChem 4000i clinical chemistry analyzer (Scil, Viernheim, Germany).

### 4.4. Histological analysis

For morphometric analysis of adrenal glands, 12 µm thick cross-sections were prepared using a cryostat (2800 Frigocut, Reichert-Jung) at −20°C and mounted on microscopic slides. Sections were fixed in 4% paraformaldehyde and stained with haematoxylin and eosin (AppliChem, Darmstadt, Germany). Images of two representative sections of each gland were taken with a photo camera (VisiCam v. 3.0, VWR, Darmstadt, Germany) connected to a binocular. Area measurements of the complete adrenal cross-section and the medullary part were performed using VisiCam software (VisiCam Image Analyzer v. 6.2.3.1, VWR).

### 4.5. *Ex vivo* dexamethasone treatment of peripheral blood mononuclear cells

PBMCs were treated *ex vivo* with DEX according to Russcher *et al*. [47] with modifications. Cells were thawed and resuspended in RPMI 1640 medium with stable glutamine (Biochrom, Berlin, Germany) supplemented with 10% FBS, 100 U ml^−1^ penicillin and 100 µg ml^−1^ streptomycin (all PAA). PBMCs were incubated in a shaking water bath for 30 min at 37°C to remove endogenous cortisol. The medium was replaced, and 3 × 10^6^ cells per well were precultured overnight (5% CO_2_, 37°C) in a 24-well plate at a density of 6 × 10^6^ cells ml^−1^. The next day, PBMCs were incubated for 4 h with increasing DEX concentrations (0, 0.5, 5, 50 and 500 nM) together with 5 µg ml^−1^ of the T-cell specific mitogen ConA (Sigma-Aldrich).

### 4.6. Gene expression analysis

Total RNA was isolated using TRI reagent (Sigma-Aldrich), DNaseI-treated (Roche, Mannheim, Germany) and cleaned using the NucleoSpin RNA II Kit (Macherey-Nagel, Düren, Germany) for tissue samples or using the RNA clean & concentrator-5 Kit (Zymo, Freiburg, Germany) for cultured PBMCs. RNA quantity and purity were determined using a NanoDrop ND-1000 spectrophotometer (NanoDrop, Peqlab, Germany), and integrity was checked on 1% denaturing agarose gels.

First-strand cDNA was synthesized using SuperScript III MMLV reverse transcriptase (Invitrogen, Karlsruhe, Germany) in a reaction containing a mixture of 500 ng random hexamers (Promega, Mannheim, Germany), 500 ng of oligo (dT)11 VN primer and 1.5 µg RNA for tissue samples or 500 ng for cultured PBMCs, according to the manufacturer's protocol.

Absolute quantification of the expression of genes of interest and reference genes (*RPL32* for tissue samples [48] and *TSC22D2* for DEX-treated PBMCs [49]) was performed using qPCR in duplicate on a LightCycler 480 System using the LightCycler FastStart DNA Master SYBRplus Green I (Roche) kit. Information on primers and amplicons is summarized in the electronic supplementary material, table S2.

### 4.7. Statistical analysis

The effect of the GR_Ala610Val_ substitution on phenotypic traits and gene expression in tissues was analysed using linear mixed models (MIXED procedure, SAS v. 9.3 software, SAS Inc., Cary, USA). The overall model included the fixed factors of GR_Ala610Val_ genotype and sex, and their interaction. The overall model further included the random effect of sire with banded main diagonal covariance structure, and the repeated effect of dam using the compound symmetry type of the block diagonal residual covariance matrix to account for genetic background. The model also included sampling order as a covariate for blood parameters and gene expression. The model included carcass or body weight as a covariate for body composition and organ morphometry, respectively. The total number of born piglets in a litter was fitted as a covariate for birth weight.

Least-squares means (LSM) of the genotype effect and their standard errors (SE) were calculated, and all pairwise differences of LSM were tested using the Tukey–Kramer procedure.

The transcriptional response of selected genes in ConA-stimulated PBMCs to DEX treatment was analysed by fitting dose-response curves using GraphPad Prism 5 (GraphPad Software, Inc., San Diego, CA).

Effects and differences were considered significant when *p* ≤ 0.05.

## Supplementary Material

Table S1. Overview of the effects of the GRAla610Val substitution on studied traits. Table S2. Overview of the effects of the GRAla610Val substitution on transcriptional responses of PBMCs to DEX ex vivo. Table S3. Oligonucleotides used in qPCR

## Data accessibility

Relevant data supporting this article have been uploaded as part of the electronic supplementary material.
